# Targeted Real-Time Assessment of Chronic Pain (TRAC-Pain) in Youth: Protocol for a Digital Biosignature Development Through a Prospective Observational Cohort Study

**DOI:** 10.2196/84781

**Published:** 2026-04-06

**Authors:** Anna H Bailes, Chi-Hung Shu, Alan Chang, Sahrish Masood, Nicole Jehl, Aliyah Davis, Jeremy Giberson, Casey Cashman, Allison Hill, Javed Gill, Ryan S McGinnis, Ellen W McGinnis, Nima Aghaeepour, Laura E Simons

**Affiliations:** 1 Department of Anesthesiology, Perioperative, and Pain Medicine Stanford University School of Medicine Palo Alto, CA United States; 2 US Pain Foundation West Hartford, CT United States; 3 Patient and Pain Advocacy Partner Palo Alto, CA United States; 4 Division of Cardiology, Heart Institute Children's Hospital of Los Angeles Los Angeles, CA United States; 5 Biomedical Engineering and Center for Remote Health Monitoring Wake Forest University School of Medicine Winston-Salem, NC United States; 6 Social Sciences and Health Policy and Center for Remote Monitoring Wake Forest University School of Medicine Winston-Salem, NC United States

**Keywords:** pediatrics, musculoskeletal pain, wearable electronic devices, biosignature

## Abstract

**Background:**

Approximately 1 in 5 children and adolescents live with chronic pain, with musculoskeletal (MSK) pain being one of the most prevalent subtypes. Unfortunately, some studies show that less than half of youth (<18 years) experience improvements with existing evidence-based treatments. Self-report measures—the current gold standard for monitoring the pain experience—are limited in their use as a single point-of-care assessment and their vulnerability to recall bias. The ubiquitous adoption of wearable technology presents a promising solution for improved monitoring of the pain experience via real-time tracking through a multisystemic lens.

**Objective:**

The purpose of this study is (1) to develop a digital biosignature of the pain experience in youth with chronic MSK pain and (2) to assess the feasibility, acceptability, and appropriateness of the captured data for future applications.

**Methods:**

All aspects of this study were designed in partnership with people with lived experience and patient advocacy partners. This is a longitudinal observational cohort study, with all study activities taking place remotely over a 12-week period. Up to 500 youth (between 14 and 24 years old) with chronic MSK pain will be enrolled through a multipronged recruitment strategy to ensure a representative sample. The participants will wear an Apple Watch throughout the study to continuously monitor physiological, sleep, and physical activity metrics. In addition, participants will complete brief “daily check-in” surveys that include gold-standard measures of the pain experience (eg, pain interference, mood, fatigue) and the option to report a “pain flare” (ie, a temporary but noticeable worsening of the usual pain symptoms). Participants will also complete a modified online Trier Social Stress Test and a 30-second sit-to-stand task to capture individual responses to standardized challenges. Traditional machine learning and deep representation learning methods will be used to develop a digital biosignature of the pain experience. The accuracy of the biosignature will be assessed through measures of model performance as compared to gold-standard self-reports.

**Results:**

This study was funded in September 2024, with data collection beginning in March 2025. As of December 15, 2025, 190 participants are enrolled, with data collection and analysis ongoing.

**Conclusions:**

This is the first study to leverage wearable health technology for real-time monitoring of the pain experience in youth with chronic MSK pain. The resulting digital end points are expected to heighten the rigor of clinical trials and provide opportunities for individually tailored interventions. The second phase of this study will investigate the implementation of “wellness alerts” triggered by abnormal smartwatch readings. Alerts would empower users toward preemptive self-management strategies, thereby enhancing self-efficacy in those living with chronic MSK pain.

**International Registered Report Identifier (IRRID):**

DERR1-10.2196/84781

## Introduction

Approximately 1 in 5 children and adolescents live with chronic pain, with musculoskeletal (MSK) pain being highly prevalent (25.7%). [1] Chronic MSK pain significantly impacts quality of life, [2] school attendance [3,4], mood [2,4], and physical function [5]. Unfortunately, despite existing evidence-based treatment methods, many children and adolescents (ie, youth) with chronic pain experience little to no reduction in pain or disability [6-8]. In fact, up to 80% of youth with chronic widespread MSK pain become adults with chronic pain [9], emphasizing the urgency of both developing improved interventions and enhancing our ability to provide just-in-time intervention drawn from existing evidence-based tools. The chronic pain experience is not consistent over time; intra- and interdaily fluctuations in symptoms and the presence of pain flares contribute to unpredictability, uncertainty, and greater impairment for youth living with chronic pain [10,11]. Unpredictable fluctuations also present unique challenges for the monitoring and treatment of chronic pain.

Self-report measures are the gold standard for monitoring the pain experience in this population [12]. These measures capture multiple components of the pain experience, including pain interference, mood, fatigue, and physical function. However, self-report metrics are often burdensome and limited by their use as a single point-of-care assessment and vulnerability to recall bias. As such, those living with chronic pain may feel frustrated by the inability to accurately portray the nature of their pain experience [13], while clinicians may be left with a lack of data to make the most effective clinical recommendations for an individual patient. This often leads to inconsistent and insufficient treatment recommendations, to increased health care utilization, and the potential for further frustration and distrust between patients and providers [14].

The nearly ubiquitous consumer adoption of health care technology—particularly wearables [15,16]—presents a promising solution for improved comprehensive monitoring of the pain experience, with opportunities for personalized medicine approaches [17,18]. Unlike self-reports, wearables provide longitudinal and objective measures of health that reflect real-time fluctuations inherent to the pain experience. Wearables also present a unique opportunity for collecting multiple biomarkers (eg, heart rate and sleep data) that can be combined with other domains to inform the development of biosignatures. Through machine learning and related analytic techniques, several digital biosignatures of human health have already been developed using data from wearable technology [17,19,20].

In summary, mechanisms underlying the persistence of chronic pain in youth remain poorly understood. Multisystem involvement (eg, autonomic, sensory, immune) and frequent symptom fluctuations complicate the reliability of traditional self-report monitoring techniques and create barriers to individualized clinical decision-making. Therefore, the purpose of this study, named Targeted Real-Time Assessment of Chronic Pain (TRAC-Pain), is to overcome the limitations of the current standards for pain monitoring through the development of a digital biosignature using consumer-available wearable technology. The expected result will be a digital biosignature of the pain experience in youth with chronic MSK pain. The secondary aim of this study is to assess the feasibility, acceptability, and appropriateness of the captured data for incorporation into future applications of the biosignature.

## Methods

### Overview

**Figure 1:**
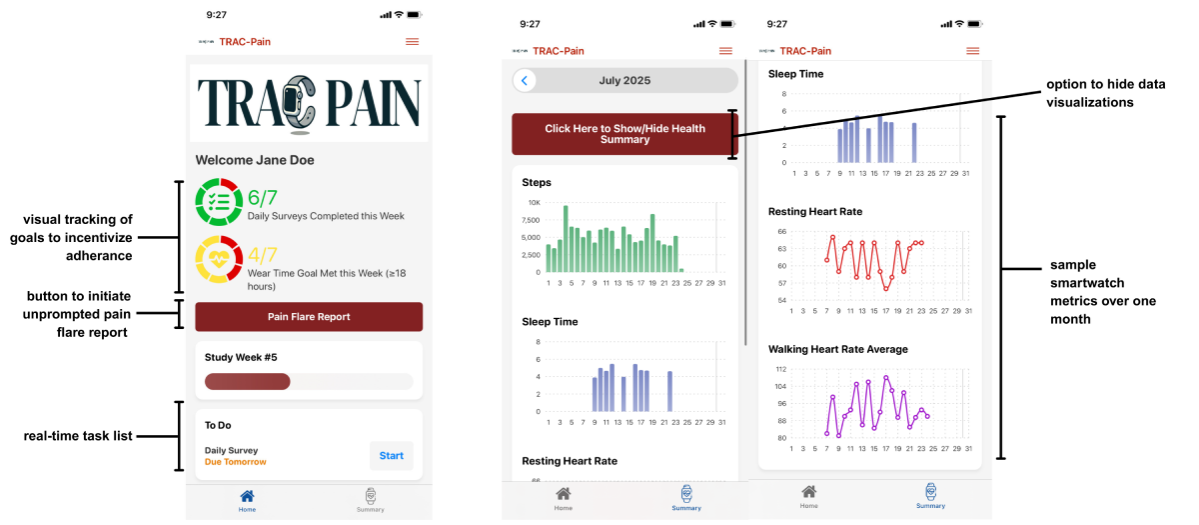
Visualization of third-party app user interface. Daily tasks are easy to identify and complete. Users can monitor their progress in the TRAC-Pain (Targeted Real-Time Assessment of Chronic Pain) study and visualize rewards when completing weekly adherence goals. The interface includes customizable options to view or hide smartwatch summary metrics.

This is a longitudinal observational cohort study, with all study activities taking place remotely over a 12-week period. Apple Health Kit (Apple Inc) and survey data will be collected and stored using a third-party digital health and research platform (Figure 1) approved by the Stanford University Institutional Review Board (IRB-77005). Study design includes multiple layers of data collection (eg, self-report measures, smartwatch metrics, and task-based measures), all of which are essential for biosignature development. Self-report measures encompass current gold-standard metrics of the pain experience and are crucial for biosignature validation. Smartwatch technology leverages the widespread adoption of wearables to capture real-time fluctuations across multiple body systems, which is critical for ensuring a holistic and longitudinal understanding of the pain experience. Task-based measures, such as the online Trier Social Stress Test and 30-second sit-to-stand task (30-s STS), are included as secondary metrics to facilitate participant comparison across standardized challenges. As understood through the biopsychosocial model of pain, multiple systems contribute to the pain experience. Therefore, it is vital to collect multiple layers of data for biosignature development. Machine learning methods are critical to the study design, facilitating an improved interpretation of multidimensional data and enhanced detection of nuanced fluctuations in pain status.

### Participants and Setting

Up to 500 youth and adolescents with chronic primary or secondary MSK pain [21,22] will be enrolled in this study. Individuals are eligible to participate if they are between 14 and 24 years old and experience MSK pain (pain in muscles, tendons, bones, joints) in one or more anatomic regions that has persisted or recurred for ≥ 3 months and is associated with significant emotional distress or functional disability. Individuals are excluded if they report only headache, orofacial, or visceral pain, have been hospitalized in the past 30 days for something other than pain, have a significant cognitive impairment that would prevent study participation (eg, severe brain injury), are currently undergoing treatment for cancer, or are pregnant or expect to become pregnant over the study period. This study is listed at ClinicalTrials.gov (NCT06867757) and preregistered on Open Science Framework (10.17605/OSF.IO/EN69C).

Participants are recruited nationwide through a multipronged recruitment strategy to ensure a representative sample. Recruitment within the Stanford health care system occurs through the Research Participation and Engagement Program (RPEP) and Stanford Children’s Health Pediatric Pain Management Clinic. Nationwide recruitment occurs through a custom-designed study website, community advertisements to pediatric multidisciplinary pain clinics in the United States, advertisements sent via postal mail, and patient advocacy networks (eg, US Pain Foundation, American Chronic Pain Association, and The Ehlers-Danlos Society). Interested participants are directed to complete a web-based study interest form as the first step of the eligibility screening. Potentially eligible participants are then contacted by the study team for additional screening based on the previously described eligibility criteria.

### Ethical Considerations

The participants—and their legally authorized representatives, if applicable—complete an IRB-approved assent/consent process before completing any study activities. All study data, including digital health information, shared medical records, and video submissions, are collected, stored, and shared according to strict confidentiality requirements of the Stanford University IRB (IRB-77005).

### Self-Report Measures (Primary)

**Figure 2:**
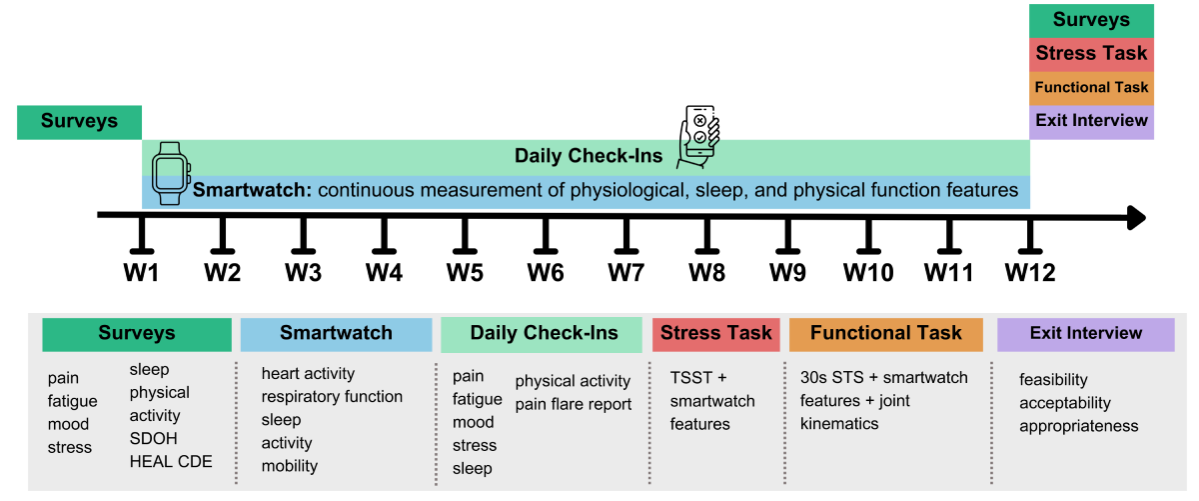
Timeline of TRAC-Pain (Targeted Real-Time Assessment of Chronic Pain) study. The study begins with surveys completed at baseline. Participants wear a smartwatch for 12 weeks and complete "daily check-in" surveys about their pain experience. At discharge, participants are invited to complete a modified online Trier Social Stress Test and 30-second sit-to-stand task (30s STS). Finally, implementation outcomes are assessed through a semistructured exit interview to guide future applications. HEAL CDE: Helping End Addiction Long Term Common Data Elements; SDOH: Social Determinants of Health.

The study design is outlined in Figure 2. Once enrolled, participants complete a battery of baseline surveys through the third-party app. Baseline surveys include self-report measures assessing pain and pain interference [23-27], fatigue [28,29], mood [30-32], stress [33], sleep [34], and physical activity [35] (Table 1).

**Table 1:** Timeline of tests and measures for the Targeted Real-Time Assessment of Chronic Pain (TRAC-Pain) study.

Domain and tests/measures					Time points administered						
					Baseline		Daily		Continuous		Discharge
**Candidate Monitoring Features (Apple Health Kit)**											
	**Physiological**										
		**Heart activity**									
			Heart rate					✔			
			HRV^a^					✔			
		**Respiratory function**									
			Respiratory rate (sleep only)					✔			
	**Physical function**										
		**Activity level**									
			Steps, bouts of activity, standing time					✔			
		**Mobility**									
			Gait speed, gait symmetry, step length, 6MWT^b^					✔			
	**Sleep**										
		Time in bed, time awake, sleep phases					✔				
**Gold Standard self-report metrics of the pain experience**											
	**Pain Interference**										
		PEG 3-item^c^			✔		✔				✔
		CALI-9^d^			✔						✔
		BPI-SF^e^			✔						✔
	**Pain**										
		PPST^f^			✔						✔
		SF-MPQ-2^g^			✔						✔
		NRS^h^ pain			✔		✔				✔
	**Fatigue**										
		PROMIS^i^-fatigue			✔						✔
		NRS fatigue			✔		✔				✔
	**Mood**										
		PHQ-2^j^			✔		✔				✔
		GAD-2^k^			✔		✔				✔
		PANAS-10^l^			✔		✔				✔
	**Stress**										
		PROMIS-stress			✔						✔
		NRS stress			✔		✔				✔
	**Sleep**										
		ASWS-SF^m^			✔						✔
		NRS sleep quality			✔		✔				✔
	**Physical activity**										
		PROMIS-Physical Function SF 8c 7-Day			✔						✔
		NRS activity level^n^			✔		✔				✔
**Natural and task-based events**											
	**Pain flare report**										
		Pain flare qualities, wellness behaviors (eg, deep breathing, pacing), daily events (eg, academic stressor, medical appointment)					✔		✔^o^		
	**Physiological response to stress task**										
		Self-report distress metrics, Apple Health Kit metrics collected during TSST-OL^p^									✔
	**Functional Task Performance**										
		Number of sit-to-stand-stand repetitions completed, joint kinematics, Apple Health Kit metrics									✔
**Additional Variables**											
	**Psychosocial, environmental, and social determinations of health**										
		PANES^q^			✔						
		mMOS-SS-8^r^			✔						
		ULS-8^s^			✔						
		Everyday Discrimination Scale – short version			✔						
		Health Care Discrimination Scale			✔						
		Children’s HealthWatch Hunger Vital Sign			✔						
		HealthBegins upstream risk screening tool			✔						
		PSS-10^t^			✔						
		CHIS^u^-language spoken at home single-item			✔						
		Demographics survey (eg, age, sex, race, gender)			✔						
	**Medical history**										
		Prior and current medical and pain history and treatments			✔						✔
	**HEAL CDE measures**										
		WHOQOL-2^v^			✔						✔
		PCS-6^w^			✔						✔
		NIDA-2^x^			✔						✔
		PGIC^y^			✔						✔
	**Implementation outcomes**										
		**Participant feedback**									
			TFA^z^							✔	
			NPS^aa^							✔	
			Exit interview							✔	
		**Adherence metrics**									
			Hours of smartwatch wear, % complete of surveys and check-ins							✔	

^a^HRV: heart rate variability.

^b^6MWT: 6-minute walk test.

^c^PEG 3-item: Pain, Enjoyment of Life, and General Activity scale.

^d^CALI-9: Child Activity Limitations Interview.

^e^BPI-SF: Brief Pain Inventory – Short Form.

^f^PPST: Pediatric Pain Screening Tools.

^g^SF-MPQ-2: Short-Form McGill Pain Questionnaire-2.

^h^NRS: Numeric Rating Scale.

^i^PROMIS: Patient-Reported Outcomes Measurement Information System.

^j^PHQ-2: Patient Health Questionnaire-2 item.

^k^GAD-2: Generalized Anxiety Disorder-2 item.

^l^PANAS-10: Positive and Negative Affect Schedule.

^m^ASWS-SF: Adolescent Sleep Wake Scale–Short Form.

^n^Not a validated measure, adapted from NRS format (ie, “On a scale from 0 to 10, how would you rate your overall physical activity today?”)

^o^Available to complete unprompted, retrospectively or in real time, outside of time-based daily check-in

^p^TSST-OL: Trier Social Stress Test, online/remote.

^q^PANES: Physical Activity Neighborhood Environment Scale.

^r^mMOS-SS-8: 8-item modified Medical Outcomes Study Social Support Survey.

^s^ULS-8: 8-item UCLA Loneliness Scale.

^t^PSS-10: 10-item Perceived Stress Scale.

^u^CHIS: California Health Interview Survey.

^v^WHOQOL-2: World Health Organization Quality of Life-2.

^w^PCS-6: Pain Catastrophizing Scale-6 item.

^x^NIDA-2: Level 2—Substance Use—Child Age 11–17 instrument from the National Institute on Drug Abuse.

^y^PGIC: Patient Global Impression of Change.

^z^TFA: Theoretical Framework of Acceptability.

^aa^NPS: Net Promoter Score.

Once the baseline surveys have been completed, the participants begin to receive “daily check-in” surveys, deployed at 5 pm (participant’s local time) and available to complete until 11:59 pm, for the duration of the 12-week study. Participants receive daily prompts through the app interface to complete these “daily check-in” surveys, which include short versions of self-report measures to quantify daily pain interference, pain, fatigue, mood, stress, sleep, and physical activity (Table 1). The daily check-in also asks participants to report whether they had a pain flare that day (“yes/no”) and to describe its characteristics. A pain-flare [10,11] is described to participants using the following language: “A temporary but noticeable worsening of your usual pain and/or symptoms (eg, fatigue, stiffness, and emotional distress) that feels more intense than what you typically deal with. Pain flares typically meet the following criteria: (1) are disruptive, (2) last longer than usual pain spikes, (3) affect more than just pain, (4) may be linked to a trigger, and (5) are bigger than normal pain changes.” Participants also have the option to initiate an unprompted retrospective or real-time pain flare report outside of the time-based daily check-in. The daily check-in also includes a daily diary for participants to note any exciting or stressful daily events (eg, academic stress, medical appointment) and any pain wellness strategies used (eg, deep breathing, pacing, journaling). The daily self-report measures are used as the gold standard (ie, ground truth) to which novel physiological markers are compared and thus are essential to ensure the clinical relevance of the biosignature.

### Smartwatch Metrics (Primary)

If participants do not have an iPhone compatible with iOS 14.1 or later and an Apple Watch compatible with iOS 15 or later, they are mailed study-provided devices (iPhone SE Gen 2, Apple Watch Series 9s) as needed. Once the devices are received, the participants are invited to enroll in the study through an app-based third-party digital health and research platform. Upon study discharge, the participants are required to return any borrowed devices. The following steps are implemented to encourage device return: (1) participants receive a prepaid return envelope and instructions for returning the device(s) upon study discharge; (2) participants explicitly agree to return device(s) as part of the consent process; and (3) frequent touch points with the study team are intended to promote good rapport and increase the likelihood of follow through on agreed returns.

The participants are instructed to wear the Apple Watch for at least 18 hours per day during the 12-week observation period. Apple Health Kit data are collected, uploaded, and stored through the third-party digital health and research platform. Metrics of interest include those related to respiratory function (eg, respiratory rate), heart activity (eg, heart rate, heart rate variability), physical activity (eg, steps, bouts of activity, standing time), sleep (eg, in-bed time, sleep phases), and mobility (eg, gait speed, walking asymmetry, step length) (Table 1). Survey completion rate and adherence to required watch wear times are monitored by the study team. Participants receive notifications from the study team to encourage high levels of adherence based on completion standards selected a priori (eg, number of daily check-ins completed each week and daily and nightly hours of wear). Participants receive weekly monetary incentives for completing each component of the study, with up to US $210 total. Those who do not meet adherence standards may not be eligible to receive a given week’s compensation. Furthermore, those consistently not meeting standards within the first few weeks of the study may be withdrawn in an effort to allocate research team time to adherent participants and prevent excessive amounts of missing data. At the end of the 12-week observation period, the participants complete an additional battery of discharge surveys, once again deployed through the third-party app (Table 1). They also complete a series of task-based measures prior to study discharge.

### Task-Based Measures (Secondary)

#### Overview

The participants complete an additional stress task and a functional task to capture physiological responses to standardized challenges. These tasks are collected on a subset of the study cohort to examine responses to acute stressors. Although not required for the development of the initial biosignature, physiological responses and performance metrics related to these standardized challenge paradigms may improve the sensitivity of the biosignature in future iterations. Additionally, the results will provide unique insight into the pain experience by allowing for a standardized comparison across participants, supporting future hypothesis generation.

#### Online Trier Social Stress Test

A modified online Trier Social Stress Test (TSST) [36] is used to measure physiological responses to an evaluative stress test known to activate the hypothalamic-pituitary-adrenocortical (HPA) system. The HPA system plays an important role in the pain experience [37]. Although originally developed for in-person testing, an online version of TSST, known as the TSST-OL, has been validated to elicit HPA activation in adolescents [36].

In the TSST-OL, participants are first shown a calming video to induce a standard neutral to positive state. Then, they are given 5 minutes to prepare a speech, as if presenting to a new class. They are instructed to describe themselves to the class and why they would be liked by other students. Next, they have 5 minutes to deliver the prepared speech, via an online conferencing platform, to a panel of neutral or critical “judges.” The judge panel will be comprised of study staff who have been trained to maintain a neutral to stern expression during the speech. After speech delivery, the participants are given another 5 minutes to complete a mental arithmetic task (ie, serial subtraction) in front of the same neutral to stern panel of judges. Participants then have a brief, unsupervised rest period prior to task debrief. Traditionally, saliva samples are collected at various points in this protocol to measure cortisol levels as a proxy for HPA activation. However, recording other physiological metrics (eg, heart rate) is more feasible in the online version of the TSST, as these metrics have been shown to fluctuate in response to the TSST [38]. Recording of physiological metrics during the TSST-OL is done via Apple Watch set to “workout” mode to ensure a high sampling rate of heart rate readings. Collecting Apple Health Kit metrics during the TSST-OL provides insights into the systemic response to emotional stressors for each participant. To mitigate psychological risk that may persist following the TSST-OL, the study team completes a participant debrief consistent with the validated TSST-OL protocol [36]. The purpose of the debrief is to further describe the reason for this task and to alleviate any participant concerns. The study’s principal investigator (author LES) is a licensed clinical psychologist and is prepared to offer additional support as needed.

#### 30s-STS Task

The 30s-STS is a measure of lower extremity strength, transitional movement mechanics, and functional exercise capacity [39,40], systems that may be impacted by chronic pain. The 30s-STS has not been validated in youth with primary chronic pain disorders. However, this task demonstrates strong psychometric properties for healthy young adults, with strong test-retest reliability (intraclass correlation coefficient=0.93) and convergent validity (Pearson *r*= –0.78 for the 5x STS and Pearson *r*=0.51 for the lateral step-down test) [40]. The 30s-STS is also useful in discriminating active versus inactive healthy young adults [40]. Therefore, the 30s-STS is expected to be an appropriate test to quantify functional performance and physiological response to physical challenge in this study population.

Participants receive explicit instructions for remote performance of the 30s-STS (video instructions in Multimedia Appendix 1). For this task, the participants are asked to perform repetitions of an STS movement for 30 seconds. They are instructed to use a sturdy chair and perform repetitions as quickly and safely as possible, while keeping their arms crossed over their chest. The participants are instructed to identify another person to act as a recorder and timekeeper for the task. This person will record the participants’ performance and ensure the test is stopped at 30 seconds. Then, the participants upload the video via the secure third-party platform for the study team to access. Established norms are available for a subset of our expected cohort (ages 18 to 24 years) and are used to evaluate participant performance (ie, number of STS repetitions) [40]. Additionally, this study assesses innovative and nuanced metrics of performance through the development and implementation of a modified human pose-estimation algorithm to estimate kinematics (eg, trunk angle, joint angular velocity) [41]. Finally, as with the TSST-OL, participants are instructed to wear their Apple Watch set to the “workout” mode throughout the duration of the 30s-STS to monitor systemic response to a standardized physical performance test.

#### Exit Interview

Following completion of the 12-week protocol, baseline and discharge surveys, TSST-OL, and 30s-STS, the participants are invited to complete a semistructured exit interview to garner feedback regarding the feasibility, acceptability, and appropriateness of the captured data. The participants also complete the Theoretical Framework of Acceptability (TFA) [42] and Net Promoter Score (NPS) [43] surveys. Feedback from the exit interview, TFA, and NPS is incorporated into the ongoing study procedures as appropriate and is particularly important for future applications of this work, specifically as a guide for implementation and validation of the digital biosignature (Phase 2).

#### Collaborations With People With Lived Experience

All aspects of this study were developed in partnership with people with lived experience, including patients, caregivers, and patient advocacy groups. These patient and advocacy partners were and continue to be involved in every aspect of the study, including grant preparation, development of recruitment materials, and selection of outcome measures. For example, based on feedback from patients and advocacy partners, the participant-facing app was modified to enable retrospective reporting of a pain flare, such as when a current pain flare is too severe to report in real time. Additionally, partner feedback prompted the research team to modify a short recruitment video that had previously focused on negative aspects of living with chronic MSK pain. The new video instead focuses on the positive impact and hope inspired by the study. Additionally, patient and advocacy partners noted that including daily visualizations of deterioration in smartwatch metrics via the app interface (eg, poor sleep, reduced activity) may exacerbate participant anxiety and hopelessness surrounding the likelihood of a pain flare. Based on this feedback, the study team modified the app interface to allow participants to hide unwanted data visualizations not required for the study. The participants can also opt out of any health app notifications or alerts for abnormal readings. Alternatively, the study team implemented positively framed visualizations and notifications through the visual tracking of smartwatch wear time goals and notifications for opportunities to implement participant-preferred wellness strategies. Importantly, patient and advocacy partners attended an in-person study launch meeting in December 2024 and continue to provide feedback on recruitment materials, outreach efforts, and research activities via virtual monthly team meetings.

### Analysis Plan

#### Outcome Measures

A digital biosignature will be developed to enable the detection of a change in pain status. The primary outcome used to validate the biosignature (ground truth) will be the participant’s self-reported onset of a pain flare. Additional models will be explored to predict secondary outcomes such as a clinically relevant change in pain interference, namely, 2 units on the Pain, Enjoyment of Life, and General Activity (PEG 3-item) scale or the onset of pain flares lasting for more than 2 days only. To improve pain flare prediction, participants may be stratified by demographic features known to impact the pain experience, and models may be adjusted using partial correlation. Accounting for individual factors may be particularly relevant when considering differences in digital access and literacy among participants residing across diverse geographic locations.

#### Data Reduction

Raw physiological signals (eg, heart activity, step counts, sleep stages) will be synchronized across modalities and aligned to a common timeline while preserving native sampling frequencies. To capture information across temporal scales, features will be engineered using a *multiresolution aggregation strategy*. For each signal, summary statistics will be computed over short windows (seconds to minutes) and further aggregated into intermediate (hourly) and daily summaries. All features derived at finer resolutions will be consolidated into a single daily feature vector, enabling the integration of short-term variability and longer-term physiological trends. The model prediction target and evaluation will be defined at a chosen time scale (eg, daily). Any windowing will only use past data relative to the prediction time to avoid leakage.

In addition to static summaries, *dynamic features* will be generated to characterize within-day temporal structure and behavior. These may include acceleration-based metrics (eg, mean acceleration), measures of temporal change (eg, slopes and rates of change), and circadian rhythm features. Complexity metrics (eg, entropy-based measures) may also be included to quantify signal regularity.

#### Handling Missing Data

Missing data due to nonwear or sensor dropout will be handled using a hierarchical approach. Short gaps will be interpolated when physiologically appropriate, whereas longer gaps will be left missing at the raw level. Daily features may be imputed using time-series–aware methods (eg, forward/backward filling within individuals). Missingness indicators will be included as additional covariates in model development to account for information loss.

#### Biosignature Development

Higher-level multivariate models will be developed, beginning with simpler traditional machine learning models before progressing to deep representation learning methods. Traditional machine learning algorithms will be trained using a nested k-fold cross-validation design. Model performance will be assessed using the area under the receiver-operator characteristic curve (AUROC), the area under the precision-recall curve (AUPRC), the *F*_1_-score, Spearman Rho, and/or Mann-Whitney tests of prediction outputs. Additionally, deep learning algorithms will be trained using train/validate/test sets where model loss, AUROC, AUPRC, *F*_1_-score, and/or Spearman Rho will be used to monitor differences between predicted outputs and true values across each epoch of training. Hyperparameter sweeps will be used to optimize the model’s performance and reduce overfitting risks. Sweeps will include adjustments to hyperparameters such as model width, model depth, learning rate, optimizer selection, learning rate schedulers, and early stopping. Model interpretability will be aided by methods such as Shapley Additive Explanations (SHAP) to identify key features relevant for clinical interpretation and application. Additionally, Phase 2 of this study is designed for prospective validation in a novel cohort, allowing for an assessment of the model’s validity beyond standard performance metrics.

#### Sample Size Justification and Power Analysis

Given that the goal of this study is not to test a specific hypothesis, but rather to iterate over an entire data set to develop higher-level multivariate models, sample size and power analysis are less relevant for our study design. However, multiple strategies will be included to ensure data sufficiency. Collecting 12 weeks of daily self-reports and continuous smartwatch-derived features for up to 500 participants will enable multiresolution feature engineering while preserving participant-level separation during model evaluation (nested cross-validation, train-validate-test splits by participant). Outcome-event counts will be monitored to reduce overfitting risk. We expect to include approximately 35 Apple Watch–derived features (eg, physiological and physical function and sleep) in our primary model, alongside demographics (eg, age, sex, social determinants of health), pain-related features (eg, pain diagnoses, medical history), and task-based performance metrics (TSST-OL, 30s-STS) in secondary models. Model complexity controls, such as regularization, feature reduction, and early stopping, will be incorporated as needed. Furthermore, our sample of up to 500 participants will far exceed those found in comparable studies [20,44]. Achieving 80% of the target recruitment (ie, 400 participants) is also sufficient based on outcome-event counts and comparable studies.

## Results

This study was funded in September 2024, with data collection beginning in March 2025. As of December 15, 2025, 190 participants have been enrolled, with data collection and analysis ongoing.

## Discussion

### Expected Findings

The purpose of this study is to develop a digital biosignature of the pain experience in youth and adolescents with chronic MSK pain. Self-report measures are the current gold standard for monitoring the pain experience and informing clinical decision-making. The purpose of developing a digital biosignature is to overcome the limitations of self-report, specifically the tendency to represent a single point in time and its inherent vulnerability to recall bias. A digital biosignature will provide real-time, objective measures of the pain experience through a holistic, multisystem lens.

This study is expected to result in several clinical benefits. First, the development and validation of digital clinical end points facilitates more robust assessment of novel therapeutics and treatment paradigms for youth with chronic MSK pain. The precise monitoring of treatment response would enhance the rigor and effectiveness of clinical trial design, leading to improved treatments, especially for those refractory to existing therapies. Second, a digital biosignature will give clinicians a deeper understanding of their patients’ individual pain experience through real-time, longitudinal monitoring of symptom fluctuations. These insights will enhance clinical decision-making and support a shift toward individually tailored interventions. Real-time tracking will also facilitate expedited shifts in a prescribed treatment plan, saving time and resources in helping patients to find the best treatment option for their specific pain presentation. Additionally, an objective biosignature may improve patients’ pain experience and interactions with clinicians by reducing the need to repeatedly recount severe pain events, an act that itself may be distressing. Finally, not only will a digital biosignature inform clinical practice, but it will also empower patients to have more control over their symptoms. Specifically, Phase 2 addresses this last point through enhanced “wellness alerts” for improved self-efficacy in living with chronic pain.

### Future Directions (Phase 2)

To further the impact of the digital biosignature that will be developed in this study, we expect to conduct a second phase of the study. The goals of Phase 2 are twofold: (1) assess clinical validation of the digital biosignature in a novel sample and (2) evaluate the accuracy of a predictive “wellness alert” system.

Phase 2 includes recruitment of a novel cohort of up to 400 youth with MSK pain. Recruitment criteria will mirror those described in this study. To ensure relevance of the digital biosignature for a representative set of adolescents, this phase will have a particular focus on recruitment of youth experiencing health disparities (eg, rural youth with limited access to tertiary pain clinics and racial and ethnic minorities). Once again, a multipronged recruitment strategy will be implemented as described in this study. Stanford Medicine’s Trial Innovation Network will provide additional support for the recruitment of youth and adolescents experiencing health disparities. Participants will use an iPhone and Apple Watch for 12 weeks to monitor physiological, sleep, and physical activity data. Devices will be provided as needed. Metrics such as AUROC, AUPRC, *F*_1_-score, positive predictive value, and negative predictive value will be used in this study phase for prospective validation of the digital biosignature. As in Phase 1, we expect that participants’ self-reported pain flare onset will be used as the primary outcome to which the digital biosignature is compared.

Participants enrolled in Phase 2 also have the opportunity to receive enhanced “wellness alerts,” which would be triggered by a deterioration in the pain experience as detected through abnormal smartwatch readings. The goal of these “wellness alerts” is to prompt users toward proactive self-management strategies (eg, pacing, sleep hygiene) in an effort to prevent or reduce the severity of the predicted pain flare. Patient and advocacy partners recommended positive framing of these “wellness alerts” (ie, opportunities for enhanced wellness strategies) as opposed to “pain flare predictions,” which may be negatively interpreted and lead to increased anxiety and hypervigilance. The accuracy of the enhanced “wellness alerts” will be assessed via comparison to self-reported pain flare onset through measures of accuracy, precision, and true positive and false negative rates. This “wellness alert” system is expected to empower patients by reducing feelings of helplessness surrounding unpredictable fluctuations in pain status. In addition, the alerts may serve as an additional tool to promote pain-related self-efficacy, which may serve as a protective factor in youth with chronic pain [45].

### Access and Dissemination

Although this study uses the Apple Smartwatch as a wearable technology of choice, the algorithms developed can be expanded across multiple monitoring options to promote broader dissemination and encourage improved access to study innovations. This study will introduce opportunities for open-access analysis tools that can be deployed across different wearable devices for improved dissemination. Furthermore, the identification of key physiological features may suggest that simpler, more economical fitness technologies (eg, pedometer or heart rate monitor chest strap) are sufficient for characterizing the pain experience in this population.

### Conclusions

The TRAC-Pain study leverages the widespread adoption of consumer wearables to overcome limitations of self-report as the current gold standard for monitoring the pain experience in youth with chronic MSK pain. This study is unique in its multipronged recruitment strategy and intentional collaboration with patient and advocacy partners from the beginning of the study design process. Clinical impacts are expected to span multiple aspects of care, impacting researchers, clinicians, and patients. In terms of research, the development of digital clinical end points will enhance the rigor of clinical trials for the development of improved therapeutics. For clinicians, a longitudinal, real-time assessment of the pain experience will introduce opportunities for individually tailored treatments. Finally, this study is an important step toward promoting patient empowerment through opportunities for proactive self-management and enhanced self-efficacy in living with chronic pain.

## Appendix

Multimedia Appendix 1 Link to 30-second sit-to-stand instructional video.

Multimedia Appendix 2 Peer review report report from the ZNS1 SRB-R (22) National Institute of Neurological Disorders and Stroke Special Emphasis Panel HEAL Initiative: Digital Biomarker (National Institutes of Health, USA).

## Abbreviations

30s-STS: 30-second sit-to-stand task
AUPRC: area under the precision-recall curve
AUROC: area under the receiver-operator characteristic curve
HPA: hypothalamic-pituitary-adrenocortical
IRB: institutional review board
MSK: musculoskeletal
NPS: Net Promoter Score
PEG 3-item: Pain, Enjoyment of Life, and General Activity Scale
SHAP: Shapley Additive Explanations
TFA: Theoretical Framework of Acceptability
TRAC-Pain: Targeted Real-time Assessment of Chronic Pain
TSST: Trier Social Stress Test
TSST-OL: Trier Social Stress Test, online/remote

## Data Availability

Data generated under this study will be made available in the Open Science Framework Registry.

## References

[1] Chambers CT, Dol J, Tutelman PR, Langley CL, Parker JA, Cormier BT, Macfarlane GJ, Jones GT, Chapman D, Proudfoot N, Grant A, Marianayagam J (2024). The prevalence of chronic pain in children and adolescents: a systematic review update and meta-analysis. Pain.

[2] Wrona SK, Melnyk BM, Hoying J (2021). Chronic pain and mental health co-morbidity in adolescents: an urgent call for assessment and evidence-based intervention. Pain Manag Nurs.

[3] Jastrowski Mano KE (2017). School anxiety in children and adolescents with chronic pain. Pain Res Manag.

[4] Miró J, Roman-Juan J, Sánchez-Rodríguez E, Solé E, Castarlenas E, Jensen MP (2023). Chronic pain and high impact chronic pain in children and adolescents: a cross-sectional study. J Pain.

[5] Wilson AC, Palermo TM (2012). Physical activity and function in adolescents with chronic pain: a controlled study using actigraphy. J Pain.

[6] Palermo TM, Law E, Zhou C, Holley A, Logan D, Tai G (2015). Trajectories of change during a randomized controlled trial of internet-delivered psychological treatment for adolescent chronic pain: how does change in pain and function relate?. Pain.

[7] Simons LE, Sieberg C, Pielech M, Conroy C, Logan D (2013). What does it take? Comparing intensive rehabilitation to outpatient treatment for children with significant pain-related disability. J Pediatr Psychol.

[8] Simons LE, Sieberg CB, Conroy C, Randall ET, Shulman J, Borsook D, Berde C, Sethna NF, Logan DE (2018). Children with chronic pain: response trajectories after intensive pain rehabilitation treatment with chronic pain: response trajectories after intensive pain rehabilitation treatment. J Pain.

[9] Kashikar-Zuck S, Cunningham N, Sil S, Bromberg MH, Lynch-Jordan AM, Strotman D, Peugh J, Noll J, Ting TV, Powers SW, Lovell DJ, Arnold LM (2014). Long-term outcomes of adolescents with juvenile-onset fibromyalgia in early adulthood. Pediatrics.

[10] Khanom S, McDonagh JE, Briggs M, Bakir E, McBeth J (2020). Adolescents' experiences of fluctuating pain in musculoskeletal disorders: a qualitative systematic review and thematic synthesis. BMC Musculoskelet Disord.

[11] Khanom S, McDonagh JE, Briggs M, McBeth J (2020). Characterizing pain flares in adolescent inflammatory and non‐inflammatory musculoskeletal disorders: A qualitative study using an interpretative phenomenological approach. Eur J Pain.

[12] Palermo TM, Walco GA, Paladhi UR, Birnie KA, Crombez G, de la Vega R, Eccleston C, Kashikar-Zuck S, Stone AL (2021). Core outcome set for pediatric chronic pain clinical trials: results from a Delphi poll and consensus meeting. Pain.

[13] Coventry J, Pacey V, Smith M, Williams CM, Ta B, Sturgiss E (2025). How children and adolescents with chronic pain describe their pain experiences: A qualitative systematic review. Patient Educ Couns.

[14] Hess CW, Rosen MA, Simons LE (2022). Looking inward to improve pediatric chronic pain outcomes: a call for team science research. Pain.

[15] Marra C, Chen JL, Coravos A, Stern AD (2020). Quantifying the use of connected digital products in clinical research. npj Digit Med.

[16] Witt DR, Kellogg RA, Snyder MP, Dunn J (2019). Windows into human health through wearables data analytics. Curr Opin Biomed Eng.

[17] Ho D, Quake SR, McCabe ER, Chng WJ, Chow EK, Ding X, Gelb BD, Ginsburg GS, Hassenstab J, Ho C, Mobley WC, Nolan GP, Rosen ST, Tan P, Yen Y, Zarrinpar A (2020). Enabling technologies for personalized and precision medicine. Trends Biotechnol.

[18] Babu M, Lautman Z, Lin X, Sobota MHB, Snyder MP (2024). Wearable devices: implications for precision medicine and the future of health care. Annu Rev Med.

[19] Ravindra NG, Espinosa C, Berson E, Phongpreecha T, Zhao P, Becker M, Chang AL, Shome S, Marić I, De Francesco D, Mataraso S, Saarunya G, Thuraiappah M, Xue L, Gaudillière B, Angst MS, Shaw GM, Herzog ED, Stevenson DK, England SK, Aghaeepour N (2023). Deep representation learning identifies associations between physical activity and sleep patterns during pregnancy and prematurity. npj Digit Med.

[20] McGinnis EW, Lunna S, Berman I, Loftness BC, Bagdon S, Danforth ChM, Price M, Copeland WE, McGinnis RS Discovering digital biomarkers of panic attack risk in consumer wearables data. medRxiv.

[21] Nicholas M, Vlaeyen JWS, Rief W, Barke A, Aziz Q, Benoliel R, Cohen M, Evers S, Giamberardino MA, Goebel A, Korwisi B, Perrot S, Svensson P, Wang S, Treede R, IASP Taskforce for the Classification of Chronic Pain (2019). The IASP classification of chronic pain for ICD-11: chronic primary pain. Pain.

[22] Perrot S, Cohen M, Barke A, Korwisi B, Rief W, Treede R (2019). The IASP classification of chronic pain for ICD-11: chronic secondary musculoskeletal pain. Pain.

[23] Krebs EE, Lorenz KA, Bair MJ, Damush TM, Wu J, Sutherland JM, Asch SM, Kroenke K (2009). Development and initial validation of the PEG, a three-item scale assessing pain intensity and interference. J Gen Intern Med.

[24] Holley AL, Zhou C, Wilson AC, Hainsworth K, Palermo TM (2017). The CALI-9: A brief measure for assessing activity limitations in children and adolescents with chronic pain. Pain.

[25] Cleeland CS, Ryan KM (1995). Pain assessment: global use of the Brief Pain Inventory. Rehabil Oncol.

[26] Simons LE, Smith A, Ibagon C, Coakley R, Logan DE, Schechter N, Borsook D, Hill JC (2015). Pediatric Pain Screening Tool: rapid identification of risk in youth with pain complaints. Pain.

[27] Melzack R (1987). The short-form McGill Pain questionnaire. Pain.

[28] Lai J, Stucky BD, Thissen D, Varni JW, DeWitt EM, Irwin DE, Yeatts KB, DeWalt DA (2013). Development and psychometric properties of the PROMIS® pediatric fatigue item banks. Qual Life Res.

[29] Quinn H, Thissen D, Liu Y, Magnus B, Lai J, Amtmann D, Varni JW, Gross HE, DeWalt DA (2014). Using item response theory to enrich and expand the PROMIS® pediatric self report banks. Health Qual Life Outcomes.

[30] Kroenke K, Spitzer RL, Williams JBW (2003). The Patient Health Questionnaire-2: validity of a two-item depression screener. Med Care.

[31] Spitzer RL, Kroenke K, Williams JBW, Löwe B (2006). A brief measure for assessing generalized anxiety disorder: the GAD-7. Arch Intern Med.

[32] Watson D, Clark LA, Tellegen A (1988). Development and validation of brief measures of positive and negative affect: The PANAS scales. J Pers Soc Psychol.

[33] Bevans KB, Gardner W, Pajer K, Riley AW, Forrest CB (2012). Qualitative development of the PROMIS(R) pediatric stress response item banks. J Pediatr Psychol.

[34] LeBourgeois MK, Giannotti F, Cortesi F, Wolfson AR, Harsh J (2005). The relationship between reported sleep quality and sleep hygiene in Italian and American adolescents. Pediatrics.

[35] Tucker C, Bevans K, Teneralli R, Smith A, Bowles H, Forrest C (2014). Self-reported pediatric measures of physical activity, sedentary behavior, and strength impact for PROMIS: item development. Pediatr Phys Ther.

[36] Gunnar MR, Reid BM, Donzella B, Miller ZR, Gardow S, Tsakonas NC, Thomas KM, DeJoseph M, Bendezú JJ (2021). Validation of an online version of the Trier Social Stress Test in a study of adolescents. Psychoneuroendocrinology.

[37] Wyns A, Hendrix J, Lahousse A, De Bruyne E, Nijs J, Godderis L, Polli A (2023). The biology of stress intolerance in patients with chronic pain—state of the art and future directions. J Clin Med.

[38] DuPont CM, Pressman SD, Reed RG, Manuck SB, Marsland AL, Gianaros PJ (2022). An online Trier social stress paradigm to evoke affective and cardiovascular responses. Psychophysiology.

[39] Jones CJ, Rikli RE, Beam WC (1999). A 30-s chair-stand test as a measure of lower body strength in community-residing older adults. Res Q Exerc Sport.

[40] Lein DH, Alotaibi M, Almutairi M, Singh H (2022). Normative reference values and validity for the 30-second chair-stand test in healthy young adults. Int J Sports Phys Ther.

[41] Boswell MA, Kidziński M, Hicks JL, Uhlrich SD, Falisse A, Delp SL (2023). Smartphone videos of the sit-to-stand test predict osteoarthritis and health outcomes in a nationwide study. npj Digit Med.

[42] Sekhon M, Cartwright M, Francis JJ (2022). Development of a theory-informed questionnaire to assess the acceptability of healthcare interventions. BMC Health Serv Res.

[43] Reichheld F (2003). The one number you need to grow. Harvard Business Review.

[44] Dudarev V, Barral O, Radaeva M, Davis G, Enns JT (2024). Night time heart rate predicts next-day pain in fibromyalgia and primary back pain. Pain Rep.

[45] Cousins LA, Kalapurakkel S, Cohen LL, Simons LE (2015). Topical review: resilience resources and mechanisms in pediatric chronic pain. J Pediatr Psychol.

