# Supplementary material for: Targeted Real-Time Assessment of Chronic Pain (TRAC-Pain) in Youth: Protocol for a Digital Biosignature Development Through a Prospective Observational Cohort Study
Source: JMIR Res Protoc. 2026 Apr 6;15:e84781. doi: 10.2196/84781 (PMC13096771; doi:10.2196/84781)
Supplement: Multimedia Appendix 1 [file resprot_v15i1e84781_app1.docx]

30-Second Sit-To-Stand Instructional Video:

<https://drive.google.com/file/d/14CTcaCg_8bGR8mWueJa33aDFkYYXlDpR/view>
