# Supplementary material for: Targeted Real-Time Assessment of Chronic Pain (TRAC-Pain) in Youth: Protocol for a Digital Biosignature Development Through a Prospective Observational Cohort Study
Source: JMIR Res Protoc. 2026 Apr 6;15:e84781. doi: 10.2196/84781 (PMC13096771; doi:10.2196/84781)
Supplement: Multimedia Appendix 2 [file resprot_v15i1e84781_app2.pdf]

1UG3NS139943-01 SIMONS, LAURA

RESUME AND SUMMARY OF DISCUSSION: This new application, submitted in response to RFA-NS-24-023 (HEAL INITIATIVE: Development and validation of remote or patient wearable device derived objective biosignatures or functional assessments to monitor pain for use as endpoints in clinical trials (UG3/UH3 - Clinical Trial Optional)), aims to transform the management of chronic musculoskeletal (MSK) pain in youth by harnessing wearable technology and advanced analytics which combines the precise physiological, sleep, and physical activity measures via wearable devices with artificial intelligence/machine learning (AI/ML) to develop and validate a monitoring digital biosignature of the individual pain experience. The review panel agreed that the impact of this study is high, given the alarmingly high issue of muscle pain in youth, and using self-measurable metrics for adolescent youth was considered significant. The review panel discussed several strengths and agreed that any data collected from this project would advance the research field. The proposed research project is comprehensive, with a clear correlation between the endpoints and a commercialization plan. The PI is highly productive and has assembled an excellent investigative team. The team includes pain physicians and experts in machine learning and device expertise, has a history of successful collaboration, is well-positioned to perform the proposed research, and includes a team management plan. Priority to identifying the patient advocate group, identifying pain flare triggers, and wellness input was considered innovative. The approach is considered transformative, well-organized, and well-described. The patient population is well-described, with a good representation of the diverse community. The UG3 stage is adequate for validating the approach and identifying the pros and cons of the system. The research environment is excellent. The milestones are well formulated and go/no-go criteria are identified. The review panel had mixed opinions on some of the approaches in the application. Some reviewers expressed that the preliminary data was derived from actigraphy, and it is unclear if the current study is also based on actigraphy. Also, how well the actigraphy data fits this patient population is unclear. However, others found preliminary data impressive and expressed that it supports the feasibility of the study. Some reviewers expressed concerns about specificity and difficulty accessing the raw data using Apple Watch to collect the data. They also questioned if these data reflect the pain state because a lot of data collected by Apple Watch overlaps with several conditions. The same reviewers also questioned how the project would match them with their targeted conditions. Moreover, how to handle the missing data when participants are not wearing the watch is not addressed. However, others disagreed and argued that the team could perform analytics from the data collected, such as respiration, sleep, and other physical activities, and link these digital endpoints to pain. They also pointed out that the participants have MyPHD and other push notifications besides recording via Apple Watch, so this is not a concern. The review panel also discussed a few minor weaknesses. The control population is unclear. The pain conditions associated with males are focused heavily, even though pain conditions affect females more. Reviewers noted that details on the sleep measures are missing, and how this sleep data is integrated into the study is unclear. The FDA consultation at the UH3 phase is also unclear. Overall, although reviewers also discussed some weaknesses, they were considered minor, and the review panel expressed a high enthusiasm about this study and rated it as outstanding to very good with high impact.

DESCRIPTION (provided by applicant): Chronic MSK pain is marked by a complex biologic response accompanied by physiological perturbance in cognition, sleep, and energy levels (fatigue), and is associated with impairments in physical and emotional function. Moreover, the chronic pain experience is not stable over time with intra- and inter-daily fluctuations and the presence of pain flares contributing to unpredictability, uncertainty, and ultimately greater impairment. Current gold standard self-report assessment is burdensome and falls short of providing comprehensive, reliable measures of the pain experience, typically reflecting single point-of-care assessment with inherent recall bias. A potential solution lies in the ubiquitous consumer adoption of wearable devices that provide a window into human health. Through artificial intelligence (AI) and machine learning (ML) several ground-breaking digital biosignatures of human health have been developed. This proposal overcomes the limitations of

self-report by combining the precise physiological, sleep, and physical activity measures via wearable devices with AI/ML to develop and validate a monitoring digital biosignature of the individual pain experience in youth with MSK pain. We are well positioned to execute UG3/UH3 aims with: (1) a highly skilled team with scientific expertise in digital technology, AI/ML, digital endpoint development, and clinical trials, clinical expertise in chronic pain in youth, and lived experience expertise from patients, caregivers, and pain advocacy groups; (2) a centralized and standardized digital data collection, processing, and storage system, the scalable and secure My Personal Health Dashboard (MyPHD), and (3) preliminary data to support our digital biosignature development capability. For UG3 phase we will enroll 500 youth (ages 14-24) with chronic MSK pain, capturing continuous physiological (heart, respiratory), sleep, and physical (activity level, mobility, gait) activity metrics via wearables with repeated intra-daily gold standard self-report of pain experience (pain interference, pain intensity, fatigue, mood, stress, pain flares). We will Incorporate user feedback on wearable use and quality of life relevance of data captured, develop a digital biosignature of the pain experience, and prepare for the UH3 phase through outreach and collaboration with (a) individuals with lived experience, (b) individuals who experience health disparities, and (c) FDA to ensure relevance, acceptability, and recruitment of underrepresented youth coupled with scalability of the algorithm for clinical use. For UH3 phase we will enroll 400 diverse youth with chronic MSK pain capturing wearable and self-report of pain experience metrics for clinical validation of the pain experience digital biosignature and accuracy of an opportunity for enhanced wellness alert system. The successful development and validation of digital endpoints are crucial for the evolution of pain management. These endpoints can advance therapeutic development by providing robust, objective measures to monitor treatment response. Our studies, supported by this RFA, will be fundamental in seeking regulatory approval for the commercialization of the associated software or for disseminating open-source analysis packages for future clinical trials.

PUBLIC HEALTH RELEVANCE: The project aims to transform the management of chronic musculoskeletal pain in youth by harnessing wearable technology and advanced analytics. It is predicated on developing a real-time digital biosignature through machine learning algorithms, which will provide an objective, comprehensive assessment of the pain experience beyond self-reporting. This endeavor not only promises to enhance patient outcomes through personalized interventions but also stands to significantly reduce the economic and emotional burden of chronic pain by enabling timely and effective treatment strategies.

CRITIQUES: The critiques of individual reviewers are provided below in an essentially unedited form. These critiques were prepared prior to the review meeting and may not have been updated or revised subsequent to the discussion at the meeting. Therefore, they may not fully reflect the final opinions of individual reviewers at the close of the group discussion or the final opinion of the group. The Resume and Summary of Discussion above summarize the final outcome of the group discussion.

CRITIQUE 1

Significance: 1
Investigator(s): 1
Innovation: 1
Approach: 3
Environment: 1

Overall Impact: The overall goal of the application is to leverage off the shelf wearables (apple watch, iPhone) to provide relevant digital health metrics that can be correlated to musculoskeletal (MSK) pain status in adolescent populations. In the UG3 phase, the investigators propose to collect observational data from a cohort of 500 youth over a 12-week period, along with user feedback. They will develop a digital biosignature based on pain status PRO data collected at specified intervals over the study

period. In the UH3 phase, the investigators propose to validate the digital biosignature developed during the UG3 phase through a 400 youth cohort over a 12-week period. After a 4-week run-in period, in addition to the same passive observational data collected during the UG3 period, the investigators will also trigger a request for a “personal wellness update” when the algorithm identifies a potential “flare” state, allowing for collection of even more specific data regarding the experience. The overall goal of the application is of high importance. Objective assessment of chronic pain intensity in youth has a high impact, and a high potential to transition to chronic pain in adulthood. Positive score-driving features for this proposal were a high significance, a strong investigator team, a robust research environment, high rigor of preliminary research, a clear and comprehensive research approach, and a high perceived innovation. A few minor weaknesses in the research approach were noted.

1. Significance:
Strengths

- The investigators are addressing a high impact and poorly understood topic of chronic musculoskeletal pain. Chronic MSK pain in adolescence is alarmingly high and may be persistent to adulthood. The proposed work could strengthen understanding in this area.
- The proposed clinical studies in the work are essential to answering the fundamental research questions posed by the investigators are well-structured to provide strong clinical impact regardless of the outcomes of the digital endpoint data collection.
- There is a high rigor in describing prior research in the application.
- The application clearly identifies correlations between the digital endpoints being collected and the potential to advance therapeutic and intervention development. It is clear that the UH3 will provide sufficient verification and validation data for acquisition of regulatory approval and commercialization.

Weaknesses

- None noted

2. Investigator(s):
Strengths

- The investigators have put together a cross-disciplinary team with relevant expertise, along with appropriate support for clinical study recruitment and a panel of lived experience experts to provide contexts for all aspects of the work.
- The PIs have the appropriate expertise, experience, and ability to organize both the technical and the clinical aspects of the project and meet milestones and timelines.
- The proposed work is directly in line with the primary expertise of the investigators, and they are well-suited for the analysis of the large datasets which will be derived from the proposed work.

Weaknesses

- None noted.

3. Innovation:
Strengths

- Primary innovations in the proposed work were identified in the topic of adolescent chronic MSK pain being investigated at scale, and in the inclusion of triggered “personal wellness updates” (potentially indicative of a pain flare but sent with the goal of not triggering an iatrogenic effect).

- Although the components were not individually viewed as highly innovative, the completeness of the ML analysis approach was viewed as innovative and increased confidence in the proposed work.
- Because of the reliance of the study on highly available and popular “off the shelf” wearables (apple watch/iPhone), the potential for transformative impact of the project on clinical practice is enhanced.

Weaknesses

- None noted

4. Approach:
Strengths

- Overall, the research approach is well-organized, clearly described, anticipates, and mitigates potential challenges, comprehensive, and provides clear benchmarks for success.
- The UG3 plan adequate addresses the selection of the specific digital measurements, includes a robust verification and validation study, along with testing, optimizing, and refining the algorithms and ensuring their accuracy and reliability in the target patient population. The plan also includes identification and evaluation of factors that may interfere with the precision and accuracy of the system.
- Additionally, the UG3 plan describes the study population for whom the endpoint is developed, the development of user-informed consent and training materials with input from individuals with lived experience and representatives from diverse communities.
- The UH3 plan effectively describes validation of the digital endpoints in the context of use against PRO data.
- The proposed milestones address study planning and team coordination, as well as the ongoing evaluation of incoming data. The specific go/no-go milestones related to successful completion of activities that demonstrate feasibility and readiness for the UH3 were seen as adequate.
- The MPI-led team management plan is robust and includes sufficient detail regarding management of all aspects of the project.
- The inclusion of patient (lived experience) experts was viewed as a strength of the proposed work.

Weaknesses

- The metrics available to the investigators through the Apple Watch/iPhone may be insufficient to capture a comprehensive view of the biopsychosocial status of the subject. The potential limitations and mitigations of this are not well-discussed.
- The plan does not specifically include provision for submission of a letter of intent to the FDA, although consultation with the FDA during the UG3 phase is noted.
- There is a lack of any noted hypothesis regarding what will be observed in the individuals during the course of the two primary clinical studies. It seems a lost opportunity to miss studying potential clusters of pain response in the context of surrounding biopsychosocial triggers.
- The research plan lacks a control population of individuals without pain which could strengthen the understanding of the uniqueness of the chronic MSK subject data derived from the proposed work.

5. Environment:
Strengths

- The research environment is excellent and is coupled with adequate institutional support, equipment, and physical resource to accomplish the proposed work.

Weaknesses

- None noted.

Protections for Human Subjects: Acceptable Risks and/or Adequate Protections

- Minimal risks to subjects anticipated
- Adequate protections against risks noted
- Direct subject benefits are minimal
- Potential scientific advance may lead to transformational changes in the discipline

Data and Safety Monitoring Plan (Applicable for Clinical Trials Only):

Inclusion of Women, Minorities, and Individuals Across the Lifespan:

- Sex/Gender: Distribution justified scientifically
- Race/Ethnicity: Distribution justified scientifically
- For NIH-Defined Phase III trials, Plans for valid design and analysis: Not applicable
- Inclusion/Exclusion Based on Age: Distribution justified scientifically
- Inclusion/exclusion criteria are clear and scientifically justified

Vertebrate Animals: Not Applicable (No Vertebrate Animals)

Biohazards: Not Applicable (No Biohazards)

Intellectual Property (IP) Strategy:

- The IP strategy is adequate, relying upon institutional technology transfer offices to make decisions, negotiate across institutions, and file appropriate patents.

Select Agents Research: Not Applicable (No Select Agents)

Resource Sharing Plans: Unacceptable

- No data sharing plan was found

Authentication of Key Biological and/or Chemical Resources: Not Applicable

Budget and Period of Support: Recommend as Requested

CRITIQUE 2

Significance: 4
Investigator(s): 2
Innovation: 4
Approach: 7
Environment: 1

Overall Impact: Combination of physiological, sleep, and physical activity measures via wearable devices with AI/ML as digital biosignatures of MSK pain in youth. For UG3 500 youth with chronic MSK pain will be enrolled, capturing continuous physiological, sleep, and physical metrics via Apple watch with repeated intra-daily gold standard self-report of pain experience. User feedback and quality of life will be collected, in preparation for the UH3 phase through outreach and collaboration with individuals with lived experience and those who experience health disparities, as well as FDA for recruitment of underrepresented youth. For UH3 400 youth will be enrolled for clinical validation and testing of a wellness alert system. Studies will be used to seek regulatory approval for the commercialization of the associated software and dissemination of open-source analysis packages for clinical trials. Strengths include 1) Team of mixed skills, and scientific expertise in digital technology, AI/ML, digital endpoint development, clinical trials, clinical expertise in chronic pain in youth, and lived experience expertise from patients, caregivers, and pain advocacy groups; 2) centralized and standardized digital data collection, processing, and storage system; 3) preliminary data (however not directly relevant). Concerns include reliance on 2 very basic equipment (Apple watch and iPhone). The biological rationale is obscure, the clinical rationale is not directly addressed, the premise that isolated metrics of pain do not account for the complexity of chronic pain is true, but the alternative 'combo' approach needs to be justified more rigorously. Apple watch data are notoriously obscure and access to raw data is prohibitive and/or problematic, in addition to other technical issues. These are key concerns that reduced the overall enthusiasm and confidence in success. Transition milestones weak on empirical metrics. Overall, the rationale of building a pain algorithm based on an Apple watch is too simplistic, no matter how robust the ML.

1. Significance:
Strengths

- Development of digital wearables for at home monitoring of MSK pain in youth, an under-represented group.
- Continuous tracking and objective prediction of pain.

Weaknesses

- The effectiveness of the project is low for future clinical trials because of the weaknesses in the approach (simplistic design relying only Apple watch, as well as weak biological and clinical rationale)
- The potential value of endpoints in advancing therapeutic development is also weak, due to the low confidence in the sensitivity of the measured obtained by the equipment.

2. Investigator(s):
Strengths

- PI Simons, clinical psychologist who evaluates and treats youth presenting with chronic pain in the Pediatric Pain Management Clinic (PPMC) at Stanford Children's Health. Ex-President, Society of Pediatric Psychology/American Psychological Association.
- Patient-oriented research translating biopsychosocial assessments into pain treatment approaches, coupled with pain neuroscience psychology.
- K24, R21, R61/33 awards.
- Working towards integrating digital tools into workflow, for e.g., applying virtual reality to address pediatric chronic pain.
- Co-PI's Aghaeepour has strong expertise in computer science and publication record.

- E McGinnis, co-founded two companies for smartphone apps in mental health, including expertise in Digital Point-of-Care Diagnostics for Childhood Internalizing Psychopathology, Digital Therapeutic for Panic Attacks, and wellness intervention.
- E McGinnis, wearable and mobile technologies with expertise in data science.

Weaknesses

- Prior work to integrate digital technology (VR) into workflow, Work in Graded Exposure Treatment (GET), are tangentially relevant and not robust enough to embark on this new, more complex journey.
- Not clear how K23 neuroscience-related studies (altered psychological processes in chronic pain via neuroimaging), fear avoidance and other research tracks are relevant to this proposal. It feels that prior experience has not been well integrated into a cohesive thesis.
- Aghaeepour's background in ML does not relate to wearables. This is a concern because digital healthcare data require specific skillset and present unique challenges.

3. Innovation:
Strengths

- Stanford MyPHD platform is a scalable, risk assessment-approved, and cost-effective software framework that can be configured and deployed to store, organize, and process different biomedical data sets, including wearables data, at large-scale, and support real-time data analysis at both the individual level and the cohort level. The system has been used to integrate streaming wearable data (e.g., heart rate, steps, sleep) for the early pre-symptomatic detection of COVID-19.
- MyPHD iOS and Android app is capable of running some of the algorithms at the phone level, while enabling e-consenting, subjective participant reporting, and other customizable surveys and displays.
- Trial Innovation Network to innovatively address critical roadblocks in clinical research and accelerate the translation of novel interventions into life-saving therapies.
- High-performance Computing Resources.

Weaknesses

- Missed opportunity that additional wearables were not incorporated, especially that the team has expertise, and the platform allows for that.
- There is limited innovation in the choice of wearables and the ML models.

4. Approach:
Strengths

- Comprehensive, longitudinal self-report assessments complemented with digital wearables.
- Centralized and standardized digital data collection, processing, and storage system.
- Preliminary data (however not directly relevant).

Weaknesses

- For the biological rationale, what is the relation between the pain condition and the features collected?

- For the clinical rationale, how to link the clinical symptoms with the digital features collected?
- The premise that isolated metrics such as pain intensity or activity level do not account for the complexity of chronic pain is true, but the alternative proposed needs to be justified more rigorously (what is the confidence that a ‘combo’ of metrics is the right approach?)
- The statement “seamless access to individual level continuous data” is misleading. Apple watch data are notoriously obscure and access to raw data is prohibitive and/or problematic. Issues with charging (daily) and sensitivity of the metrics were overlooked.
- There is a missed opportunity to check whether self-reports collected would be enough to train an accurate ML algorithm, even without the incorporation of digital technology.
- Will subjects adhere to daily input of a battery of self-reports over long periods? How will missing data and entries be handled?
- What is the direct relevance of prior digital tools to the current proposal?
- Some transition milestones are narrative and not numerical.

5. Environment:
Strengths

- The Stanford University Department of Anesthesiology, Perioperative and Pain Medicine has outstanding clinical facilities, a premier center for patient care, teaching and research
- Dedicated wet lab research space, and space to human pain research
- The Pediatric Pain Management Clinic (PPMC) Stanford Children's Health Specialty Services at Stanford Children's Health, the Lucile Packard Children's Hospital Stanford and Stanford Children’s Health Pain Rehabilitation Center are all dedicated to pain in infants, children, and adolescents.

Weaknesses

- None noted

Study Timeline
Strengths

- Detailed description of timeline, enrollment, and resources (CTSA) with justification.

Weaknesses

- None noted.

Protections for Human Subjects: Acceptable Risks and/or Adequate Protections
Data and Safety Monitoring Plan: Acceptable

Inclusion of Women, Minorities, and Individuals Across the Lifespan:

- Sex/Gender: Distribution justified scientifically
- Race/Ethnicity: Distribution justified scientifically
- For NIH-Defined Phase III trials, Plans for valid design and analysis: Scientifically acceptable
- Inclusion/Exclusion Based on Age: Distribution justified scientifically

Vertebrate Animals: Not Applicable (No Vertebrate Animals)

Biohazards: Not Applicable (No Biohazards)

Intellectual Property (IP) Strategy: Adequate and elaborate

Select Agents Research: Not Applicable (No Select Agents)

Resource Sharing Plans: Unacceptable

- Missing

Authentication of Key Biological and/or Chemical Resources: Not Applicable

Budget and Period of Support: Recommend as Requested

CRITIQUE 3

Significance: 2
Investigator(s): 1
Innovation: 4
Approach: 3
Environment: 1

Overall Impact: Dr. Simons and team have a well-established infrastructure for developing and testing digital solutions. This reviewer was impressed by the overall organization and structure of the grant application. They are deploying the Apple Watch, a popular (esp. among adolescents), consumer-grade digital device to quantify respiratory activity, heart activity, sleep, physical activity (level, mobility, gait), and weather. They plan to associate these digital measures with "gold standard" monitoring metrics for pain conditions. It was admirable that they plan to collect a socioeconomically diverse sample in a previously underserved population: adolescents with chronic pain conditions. Of course, recruiting and retaining pediatric msk patients would seem to be complicated by many factors; but this reviewer was fully persuaded by their detailed recruitment strategy which drew from 8 local and national resources and by their retention strategy which included infographics, a study webpage, and tailored notifications via the MyPHD app and text/email reminders. The gratuitous listing of past projects (in surgical recovery, pregnancy, fall risk, LEMURs, etc.) detracted from the application's main point: the team has ample experience conducting digital health studies, and are now turning their infrastructure to solve a new problem: pediatric chronic pain. This space could have been used to develop the methods section more deeply, helping reviewers understand exactly which types of analyses would be done with which specific measures. The lack of a clear, detailed methods section was a significant weakness of this grant application. Another example of unnecessary, gratuitous space filling is the reproduction of "Table 1. Tests and Measures" in both the Approach and Study Procedures. Applicants should consider that, in a review scenario, a shorter grant is preferred to a longer grant as long as it articulates clearly the necessary, persuasive information. Such as a clear methods section. In addition, the applicants could have made clearer that they were actually proposing a multi-modal (multiple wearable sensors), multi-instrument (wearable + smartphone) data collection protocol with redundancies (i.e., if one component failed, there were other data streams to be explored). As someone sympathetic to this type of project, this could have been made clearer to persuade/inform less familiar reviewers. Overall, this is a well-thought project, highly technical methods, and a reasonable contribution to the literature. Critically, the question is "Will this work?" It was reassuring that the 2-phase structure of this grant mechanism, combined with clear, and measurable go/no-go milestones reflects a conservative approach that will give this team a reasonable opportunity to accomplish something that promises to be of benefit to pediatric patients musculoskeletal pain conditions.

1. Significance:
Strengths

- The goal of this project is to (UG3) develop and (UH3) validate/verify a monitoring digital biomarker profile endpoints in pediatric chronic pain conditions that might be useful in future clinical trials. The proposal persuasively argues that individual response monitoring (potentially) lies in the ubiquitous adoption of digital health. The proposal further includes FDA consultation about regulatory approval, although it remains unclear to me what the nature of this consultation will be. All aspects of this proposal seem directly in response to the NOFO.
- The application was well supported by an impressive amount of preliminary data in other disease groups—fall risk, childhood mental health, pregnancy, panic—but not pediatric chronic pain. Given this, this team was well positioned to move into the chronic pain space, especially given the PI's training as a pain psychologist. In fact, one wonders why they had not entered this space before.
- In the UH3 phase, this reviewer liked the involvement of patient advocacy groups in the development of positive/affirming measures check-ins. This seems like a thoughtful and productive arrangement for all parties.

Weaknesses

- An aspect of the grant remains unclear: if many studies have already shown that pain is related to decreased mobility, emotional state, and sociability and, if we further can assume (based their prevalence, usage, etc.) that digital devices accurately (or accurately *enough)* measure mobility, emotional state, and sociability, then why do we need to combine these variables into a single pain digital biomarker profile? This aspect of the grant could had been fleshed out more.
- The specific role of how variability in the digital assessment might connect with (or change) treatment remains unclear. When reading through the UG3 aims, it was anticipated the "so what?" to come through in UH3 phase. However, the "trigger alert" and the "enhanced wellness alert" system was the only change I could detect. Given the PI's work as a psychologist, one might have anticipated targeted content to be pushed to the patient. EG: if deterioration of a patient's sleep is detected, the app might push that patient CBT sleep or some such change-specific content. This would be more than an alert system (an iPhone currently tells when one hasn't slept well, or hasn't walked enough steps), but a treatment device.
- It was not clear how specifically the project was going to seek FDA approval, outside what they described as a consultation with the FDA.

2. Investigator(s):
Strengths

- Reasonable split among the Co-PIs. Simons will do clinical things. Aghaeepour will do the analytics.
- The application convinces that the investigators were well equipped to study this population (peds), with these tools (digital phenotypes, algorithm development, in this timeframe (2□3 years). The PI (LS) is a superhuman, current K24 and R33 awardee who has been prolific in this space. She has run clinical trials (randomized controlled trial published Pain 2024). Her K24 focuses on Co-design methods (featured in Lancet Child/Ad). And she has ample experience with digital tools. The Co-PI (NA) is also an accomplished data scientist with a superb track record of collaboration and algorithm development. Other members as equally compelling. Co-Is include Ellen & Ryan McGinnis, who are equally compelling.
- The goals of the project seem well suited to the expertise of the team.

Weaknesses

- No concerns.

3. Innovation:
Strengths

- Well-designed study which would be useful for pediatric pain patients in the long-term.

Weaknesses

- It wasn't clear why, if these digital tools have been tested in multiple other cohorts, they needed to be validated in this pediatric population. One might assume that the intervention (not the digital biomarker) would be peds-specific, and it is somewhat surprising that the application did not pair a digital measurement with a digital intervention—at minimum to establish feasibility in the UH3 phase.

4. Approach:
Strengths

- UG3 Phase: define peds msk pain digital biosignature.
- UH3 Phase: validate peds msk pain digital biosignature.
- The team does an excellent job of including patient advocacy groups and has, through the K24, ample experience with co-design methodologies.
- Milestones—outside the FDA milestone (described below) all other milestones seem reasonable.
- Team Management Plan. This seemed very reasonable and an ideal collaborative effort.
- Data Management and Statistical Analysis: no concerns about this team's data management plan. They will further apply a previously developed series2signal methodology to ped chronic pain. This reviewer further like the semi-supervised approach to allow for more interpretability of the model. The further disregard for physician diagnosis as a health equity point is compelling—one'd never considered that youth with medical mistrust might be misdiagnosed, though doesn't seem unreasonable (if there's evidence to support this?).

Weaknesses

- The term "AI/ML" seems appropriate in a press release, but not in a methods section. AI is a general class of algorithms. It is unclear what exactly the investigators want to predict in UG3 Aim 2 and UH2 Aim 1. UG3 Aim 2 states: "Individual candidate monitoring features (Table 1) will be used as inputs to longitudinal deep learning models trained to predict gold standard monitoring metrics as well as natural and task-based pain outcomes in either single- task or multi-task model training settings." And, in Table 1, there are 7 rows of "Gold Standard Monitoring Metrics" with, 14 different clinical scales. But it's unclear how this translates to prediction, precisely. For example, UH3 Aim 1 proposes to validate the UG3 Aim 2 biomarker "with a particular focus on the metrics of AUROC, AUPRC, F1 score, positive predictive value, and negative predictive value." It's unclear whether there will be, say, 14 separate ROC curves. If it would make more sense to pool the 14 clinical scales and, perhaps with a PCA, extract a latent structure representing all 14. This might then be able to serve as a summary "gold standard" measure against which the digital biomarkers can be compared.

- The structure of the “FDA consultation” further remains unclear to me. Is this a service that the FDA provides? The authors state that “through consultation with… (c) FDA, we will ensure relevance, acceptability, and recruitment of underrepresented youth coupled with scalability of the algorithm for clinical use.” Some version of this phrase is repeated a few times throughout the grant, but it’s unclear what this means exactly. But would like to know. why the applicants didn’t undergo a “pre-submission” consultation with the FDA, so as to not have to (possibly) change their protocol based on the same consultation.
- One of the criteria of this grant mechanism was that “Assessing how the digital endpoint or functional/behavioral assessment responds to therapeutic or behavioral interventions by determining whether the digital assessment can accurately measure changes in the targeted health outcomes or behaviors in response to specific interventions.” There isn’t seemed to be any specific method to trace how the project’s proposed digital biomarker might change with specific, targeted health outcomes.

5. Environment:
Strengths

- Excellent Facility/Environment statement. No concerns at all that these experienced researchers at Stanford can execute the various aims/milestones of this proposal.

Weaknesses

- None noted

Protections for Human Subjects: Acceptable Risks and/or Adequate Protections

- Although this section was a rehashing of the Approach, they adequately convinced they had security measures in place to protect participants. Given adolescent population, parents will be present for consent/assent protocol.

Data and Safety Monitoring Plan (Applicable for Clinical Trials Only):

Inclusion of Women, Minorities, and Individuals Across the Lifespan:

- Sex/Gender: Distribution justified scientifically
- Race/Ethnicity: Distribution justified scientifically
- For NIH-Defined Phase III trials, Plans for valid design and analysis: Not applicable
- Inclusion/Exclusion Based on Age: Distribution justified scientifically
- This is an excellently considered Recruitment/Retention plan.

Vertebrate Animals: Not Applicable (No Vertebrate Animals)

Biohazards: Not Applicable (No Biohazards)

Intellectual Property (IP) Strategy:

- IP Strategy: Tell Stanford's OTL. But what does Wake Forest's Innovation office require? Seems unlikely that Wake Forest will give away their IP.
- This reviewer enjoyed the market landscape analysis by BBC Research.

Select Agents Research: Not Applicable (No Select Agents)

Resource Sharing Plans: Acceptable

Authentication of Key Biological and/or Chemical Resources:

- Not Applicable (No Relevant Resources)

Budget and Period of Support: Recommend as Requested

CRITIQUE 4

Significance: 3
Investigator(s): 3
Innovation: 4
Approach: 4
Environment: 3

Overall Impact: This UG3/UH3 application from MPI investigators at Stanford University and Wake Forest University to develop a digital biologic signature using AI and machine learning for youth ages 14 to 24 with chronic musculoskeletal pain (MSK) is considered. The problem of MSK pain is considered significant in terms of costs and quality of life. Identification of a marker beyond self- report is needed to develop a valid and reliable outcome measure. The research team brings expertise in digital biologic prediction in the various patient groups as well as the chronic pain in pediatric populations. The UG3 component will involve 500 participants primarily recruited from Stanford Health Center while the UH3 component will recruit 400 participants with a particular strategy for rural and underserved groups. The study will be housed at Stanford University with its rich technology and pediatric pain infrastructure including a CTSA which will assist with recruitment of participants. The UH3 component will be informed by UG3 findings as well as consultation from patient groups and diversity experts. The investigative team appears to have the expertise needed to conduct the study. There are many strengths to the proposed approach including the study timeline, preliminary data from other patient groups where biological digital signatures have been developed and tested, the use of tools both in English and Spanish, attention to physical mobility characteristics such as gait, and long-term plans involving the technology transfer and FDA approval. The challenges and potential pitfalls are addressed. PROMIS measures will be used for the purpose of aligning digital signature with the gold standard. Despite the many strengths of the project there are several concerns. The definition of MSK pain which may be self-report of one or more chronic pain episodes, the wide age range of the participants, the retention of subjects and the possibility of significant missing data, failure to address the consequences of having a predominantly girl/women population, and some aspects of the preliminary data presentation.

1. Significance:
Strengths

- The focus on chronic musculoskeletal (MSK) pain in adolescence and young adults is significant based on known reduced quality of life indicators and health care costs. Wearable devices will provide data that may help predict or explain pain episodes.
- Real time assessment of a complex disorder may provide new insights to potential therapeutic approaches.
- The combination of both physiological and emotional response signals is unique in this complex disorder.
- The potential of the project to move beyond self-report assessment of pain. The inclusion of factors including sleep and physical activity in addition to self-report of pain.
- The use of a commercially available technology, the Apple Watch. And the Personal Health Dashboard.

- The goal of creating a scalable wearable device approach that can be broadly applied
- The emphasis on under-represented groups as well as engagement of patient advocacy groups.
- Stanford University has infrastructure in place that should facilitate the transfer or facilitate the regulatory outcomes.

Weaknesses

- The preliminary data while impressive in the range of patient groups that the investigators have created biologic digital programs, do not provide confidence with regard to participant recruitment and retention. The results of several studies were challenging to interpret.
- The project has the ability to collect a massive amount of data from which to test with the proposed a model, its deployment is less clear.

2. Investigator(s):
Strengths

- The PI of MPI project Laura Simons is a clinical psychologist and Professor in the Department of Anesthesiology, Perioperative, and Pain Medicine. She currently has a K24 and a research record in the field of pediatric pain assessment. Nima Aghaeepour is a PhD in bioinformatics will manage the digital endpoint development. Dr. Aghaeepour has machine learning work expertise related to immune modeling in various patient groups. Eileen McGinnis is Assistant Professor of Public Health Sciences and has a PhD in clinical psychology. The team has expertise in development of Apps linked with accessible wearable sensors and smartphones to measure psychological mood symptoms. Dr. McGinnis will advise on remote data collection, data analysis, visualization, and documentation. Ryan McGinnis has a PhD in mechanical engineering and other training related to technology commercialization and is Director of the Center for Remote Patients participant monitoring at Wake Forest University. He will serve as the Wake Forest University site lead.
- A team management plan is in place. The team also includes members will expertise related to underserved populations.

Weaknesses

- There is no clinical scientist with expertise related to MSK pain.
- There is a plan for an annual team meeting and meetings as needed to address challenges with recruitment and retention. A more structured plan would have been ideal.

3. Innovation:
Strengths

- If found that measures such as respiration, heart rate, heart rate variability, sleep (including REM?) can be used to identify a flare that has the possibility of being useful. The innovation of the proposed study is integration of self-report with the biological measures which may be helpful for a group of adolescents and young adults.

Weaknesses

- It is not clear whether an approach that uses an Apple watch is economical although the investigators do address the need to consider other devices that may be used in the future.
- A concern is that the approach is rather complex and is potentially burdensome that it may limit the ability to reliably predict a flare.

4. Approach:
Strengths

- The investigative team conducted preliminary studies in particular the PanicMechanic to test in individuals with repeated panic attacks and followed procedures similar to that of the proposed study. Other groups studied include patients recovering from surgery, fall risk, and pregnancy monitoring and preterm delivery. These studies provide proof of concept for UG3.
- The plan for the UG3 to validate the algorithm for UH3 and to plan for recruitment strategies for rural and underserved population recruitment.
- For the most part there is consideration given to potential limitations with the approach.
- The longitudinal design for UH3 is a strength – if the participants are willing as demonstrated in UG3 to wear the device and complete questionnaires as planned.
- The recruitment of participants uses resources of Stanford University CTSA.
- There is evidence that the team can deploy wearables and that in several cases predict events or outcomes. The plan to expand to patients with lupus and sickle cell anemia.
- The milestones are articulated.

Weaknesses

- The preliminary studies are challenging to interpret. While associations were found between the digital signals and outcomes, the precision of the findings was not clear.
- The preliminary studies were not conducted in a population of adolescents and young adults. For example, what determines that a ‘flare’ has occurred. There is no specific hypothesis, e.g., does poor sleep the day before increase the likelihood of greater pain the next day.
- The rationale for the tasks, i.e., Trier stress test and physical test, is not clear. Both the flare and report of pain are self-assessments. The rationale for not using medical diagnosis needs better support.
- The fact that 80% of the sample is likely to be female may be due to additional factors beyond fibromyalgia. Whether endometriosis, dysmenorrhea, and abdominal pain which co-occur often with MSK pain might be an important consideration was not addressed.
- There was inadequate presentation of preliminary data related to prior experiences in recruiting a diverse population.
- What is the definition of rural that will be used.
- The inclusion/exclusion criteria are rather broad.
- It may be overly optimistic to assume based on understanding of MSK and variety of factors involved in the pathophysiology that flare pain can be predicted. As noted by the investigators the use of strategies such as ‘nudging’ may be useful.
- What are the potential thresholds that will be used for sleep and pain.

5. Environment:
Strengths

- The environment at Stanford University Department of Anesthesiology, Perioperative and Pain Medicine has both clinical facilities and human research space. The Stanford MyPHD platform is scalable and secure framework.
- Unique aspects at Stanford University include the CTSA with Trials Innovation Network the Collaborative Health Outcome Information Registry that was developed to collect qualitative information on patients in the Stanford Pain Management Center, and Biobehavioral Pediatric Pain Lab.

Weaknesses

- None noted.

Study Timeline:
Strengths

- The study will utilize resources of the Stanford CTSA.

Weaknesses

- The ability to recruit rural and underserved populations for the UG3 is a concern.

Protections for Human Subjects: Acceptable Risks and/or Adequate Protections

- The study will involve the recruitment of individuals ages 14-24. Subjects will be enrolled based on self-report of MSK.

Data and Safety Monitoring Plan (Applicable for Clinical Trials Only):

Inclusion of Women, Minorities, and Individuals Across the Lifespan:

- Sex/Gender: Distribution justified scientifically
- Race/Ethnicity: Distribution not justified scientifically
- For NIH-Defined Phase III trials, Plans for valid design and analysis:
- Inclusion/Exclusion Based on Age: Distribution not justified scientifically
- The sample is likely to be 80% female and 30% Hispanic for both G3 and H4. Efforts to recruit a more diverse sample for H4 were described. There is no attention to co-morbid conditions which are likely to be more common in females. The justification for the age range is not strong.

Vertebrate Animals: Not Applicable (No Vertebrate Animals)

Biohazards: Not Applicable (No Biohazards)

Intellectual Property (IP) Strategy:

- The Office of Technology Licensing at Stanford will be used. This office has a systematic plan. The application states that Stanford will work closely with Wake Forest.

Select Agents Research: Not Applicable (No Select Agents)

Resource Sharing Plans: Acceptable

Authentication of Key Biological and/or Chemical Resources: Not Applicable

Budget and Period of Support: Recommend as Requested

THE FOLLOWING SECTIONS WERE PREPARED BY THE SCIENTIFIC REVIEW OFFICER TO SUMMARIZE THE OUTCOME OF DISCUSSIONS OF THE REVIEW COMMITTEE, OR REVIEWERS' WRITTEN CRITIQUES, ON THE FOLLOWING ISSUES:

Protections for Human Subjects: ACCEPTABLE . Adequate protections are in place for the protection of the human subjects in the study.

Inclusion of Women Plan: ACCEPTABLE Males and females are included in the study at scientifically appropriate levels.

Inclusion of Minorities Plan: ACCEPTABLE This study includes adequate minority representation.

Inclusion Across The Lifespan Plan: ACCEPTABLE Only participants between the age of 14-24 will be included in the study, and this is appropriate given the research topic

COMMITTEE BUDGET RECOMMENDATIONS: The budget was recommended as requested.

Footnotes for 1UG3NS139943-01; PI Name: SIMONS, LAURA E

NIH has modified its policy regarding the receipt of resubmissions (amended applications).See Guide Notice NOT-OD-18-197 at https://grants.nih.gov/grants/guide/notice-files/NOT-OD-18-197.html. The impact/priority score is calculated after discussion of an application by averaging the overall scores (1-9) given by all voting reviewers on the committee and multiplying by 10. The criterion scores are submitted prior to the meeting by the individual reviewers assigned to an application, and are not discussed specifically at the review meeting or calculated into the overall impact score. Some applications also receive a percentile ranking. For details on the review process, see http://grants.nih.gov/grants/peer_review_process.htm#scoring.
